# *CDKAL1* gene rs7756992 A/G and rs7754840 G/C polymorphisms are associated with gestational diabetes mellitus in a sample of Bangladeshi population: implication for future T2DM prophylaxis

**DOI:** 10.1186/s13098-021-00782-w

**Published:** 2022-01-28

**Authors:** U. S. Mahzabin Amin, Nahid Parvez, Tahia Anan Rahman, Md. Rakibul Hasan, Keshob Chandra Das, Sharmin Jahan, Muhammad Abul Hasanat, Zeba I. Seraj, Md. Salimullah

**Affiliations:** 1 Molecular Biotechnology Division, National Institute of Biotechnology (NIB), Ganakbari, Ashulia, Savar, Dhaka, 1349 Bangladesh; 2 Department of Endocrinology and Metabolism, Bangabandhu Sheikh Mujib Medical University (BSMMU), Dhaka, Bangladesh; 3 Department of Biochemistry and Molecular Biology, University of Dhaka, Dhaka, Bangladesh

**Keywords:** GDM, *CDKAL1*, rs7756992 A/G, rs7754840 G/C, SNP, T2DM

## Abstract

**Background:**

Association of single nucleotide polymorphisms (SNP) rs7756992 A/G and rs7754840 G/C of cyclin-dependent kinase 5 regulatory subunit-associated protein 1-like 1 (*CDKAL1*) gene with the susceptibility of gestational diabetes mellitus (GDM) has been studied in a group of Bangladeshi women.

**Methods:**

In this case–control study, 212 GDM patients and 256 control subjects were genotyped for rs7756992 and rs7754840 by PCR-RFLP and TaqMan™ allelic discrimination assay method respectively. Genotyping results were confirmed by DNA sequencing and replicated TaqMan™ assay. The odds ratios and their 95% confidence interval were calculated by logistic regression to determine the associations between genotypes and GDM.

**Results:**

The genotype frequencies of rs7756992-AA/AG/GG in the GDM group and the control group were 37%/48%, 53%/45%, 10%/7% and those of rs7754840-CC/CG/GG were 51%/55%, 40.1%/39.8%, 9%/5% respectively. Under dominant and log additive models rs7756992 was revealed significantly associated with GDM after being adjusted for family history of diabetes (FHD) and gravidity. Conversely, rs7754840 was significantly associated (*P* = 0.047) with GDM only under the recessive model after the same adjustment. The risk allele frequency of both SNPs was higher in the GDM group but significantly (*P* = 0.029) increased prevalence was observed in the rs7756992 G allele. When positive FHD and risk alleles of these SNPs were synergistically present in any pregnant woman, the chance of developing GDM was augmented by many folds. The codominant model revealed 2.5 and 2.1 folds increase in odds by AG (rs7756992) and GC (rs7754840) genotypes and 3.7 and 4.5 folds by GG (rs7756992) and CC (rs7754840) genotypes respectively. A significant 2.7 folds (*P* = 0.038) increase in odds of GDM resulted from the interaction of rs7756992 and family history of diabetes under the dominant model. The cumulative effect of multigravidity and risk alleles of these SNPs increased the odds of GDM more than 1.5 folds in different genotypes.

**Conclusion:**

This study not only revealed a significant association between rs7756992 and rs7754840 with GDM but also provided the possibility as potential markers for foretelling about GDM and type 2 diabetes mellitus in Bangladeshi women.

**Supplementary Information:**

The online version contains supplementary material available at 10.1186/s13098-021-00782-w.

## Introduction

Gestational diabetes mellitus (GDM) is varying degree of carbohydrate intolerance that is first recognized during pregnancy and is a common obstetric complication. An impaired compensatory increase in insulin secretion to overcome the pregnancy-induced insulin resistance characterizes this disease. Various reports indicate the risk of developing Type 2 Diabetes Mellitus (T2DM) later in the life of individuals having GDM and it notably influences the metabolic health of their offspring both in the short and long run [1]. Yet, the metabolic pathways involved with this complication need to be better understood.

This complication (GDM) is rising rapidly in the prevalence of 36.6% of total pregnancies in Bangladesh [2, 3]. This prevalence varies from 0.7 to 51% in Asia [4–7]. Differences in ethnicity, diagnostic criteria, screening procedures, and population characteristics may account for the large discrepancy in prevalence rates [7]. This disease has been known to have a genetic basis [8, 9] but remarkably few susceptibility genes with strong and reproducible effects have been identified so far. Compared to T2DM, the genetics of GDM has been less studied [10, 11]. However, a concordance of risk alleles, as well as the direction of their effect has been reported. Up to date, only one genetic marker of GDM has been reported in Bangladesh [12, 13].

*CDKAL1* gene is located on chromosome 6p22.3 and encodes a 65-kD protein CDKAL1 which may be involved in beta cell dysfunction and T2DM susceptibility [14]. Evidence of this protein functioning also as a tRNA modification enzyme has been shown in some recent studies and activity of which is associated with ATP generation [15–17]. Downregulation of *CDKAL1* expression was assumed to increase the activity of cyclin-dependent kinase 5 (CDK5) [18]. So CDK5 induced insulin secretion might be regulated by binding of CDKAL1 to the CDK5 activator p35 [19–21]. However, further investigations need to state exactly how CDKAL1 alters insulin release in pancreatic beta cells and the susceptibility to T2DM by interactions between these proteins [22]. As T2DM and GDM share similar pathophysiological backgrounds [23] there must be some common genetic variants between these two disease entities. However, variations in insulin release, pancreatic cell functions, hemoglobin A1C level, and/or response to pancreatic KATP channel agonists [21, 24, 25] as well as T2DM, GDM, ulcerative colitis, Crohn’s disease, obesity, and/or birth weight [19, 26–28] have been reported to be associated with some intron variants; rs7754840, rs7756992, and rs10946398 of this gene. The major allele G of rs7756992 of *CDKAL1* is conferred a higher risk of T2DM [20]. This SNP was also found to be associated with impaired insulin secretion [19, 29] and diabetic retinopathy [30]. In pregnant women, rs7754840 conferred variations in glucose induced GIP (glucose-dependent insulinotropic polypeptide) response [31].

In the context of the situation, the present study aims to investigate the association of *CDKAL1* gene polymorphism rs7756992 A/G and rs7754840 G/C with GDM in a group of Bangladeshi pregnant women. This is the first study to evaluate the genomic variation of these SNPs of GDM candidate genes for the Bangladeshi population compared with other ethnic groups.

## Methods and materials

### Study subjects

This case control study encompassed 468 unrelated pregnant women irrespective of trimester attending an antenatal clinic for their routine treatment and checkup at the Department of Gynecology and Obstetrics, Bangabandhu Sheikh Mujib Medical University (BSMMU). Individuals with known diabetes mellitus, acute critical illness, steroid treatment due to any cause, established organ dysfunction e.g., chronic kidney disease, chronic liver disease, heart failure, known case of thyroid dysfunction, were excluded. Informed consent was taken from participants and the research ethics committee (REC) of the National Institute of Biotechnology (NIB) approved the study protocol (NIBREC 2016-04).

### Body mass index

Anthropometric measurements of weight and height were obtained by standardized techniques. The body mass index (BMI) was calculated as the weight in kilograms divided by the square of the height in meters. BMI was measured in both GDM patients and normoglycemic pregnant controls.

### Oral glucose tolerance test (OGTT)

Participants underwent 75 gm OGTT on the appointed day after 08–10 h overnight fasting and glycemic status determined using WHO 2013 criterion for GDM [fasting plasma glucose (FPG) 5.1–6.9 mmol/L and/or 1-h plasma glucose (OPG) ≥ 10.0 mmol/L and/or 2-h plasma glucose (TPG) 8.5–11.0 mmol/L]. Plasma glucose was assayed by the glucose-oxidase method using commercial kits in an automated analyzer (Dade Behring, Germany). If the glycemic status was found normal when tested before the 24th week of gestation, the mother was advised to repeat the OGTT during 24th to 28th week of gestation, and glycemic status was reconsidered. Otherwise, they were excluded from this study.

### DNA extraction

Genomic DNA was extracted from peripheral blood samples of patients and controls using PureLink^®^ Genomic DNA extraction kit (Invitrogen) according to the manufacturer’s protocol. The purity and concentration of the extracted DNAs were tested with NanoDrop 2000 UV Vis Spectrophotometer. DNA samples with an OD260/OD280 ratio between 1.8–2.0 and concentrations more than 80 ng/mL were used for genotyping.

### Polymerase chain reaction-restriction fragment length polymorphism (PCR-RFLP)

**Fig. 1:**
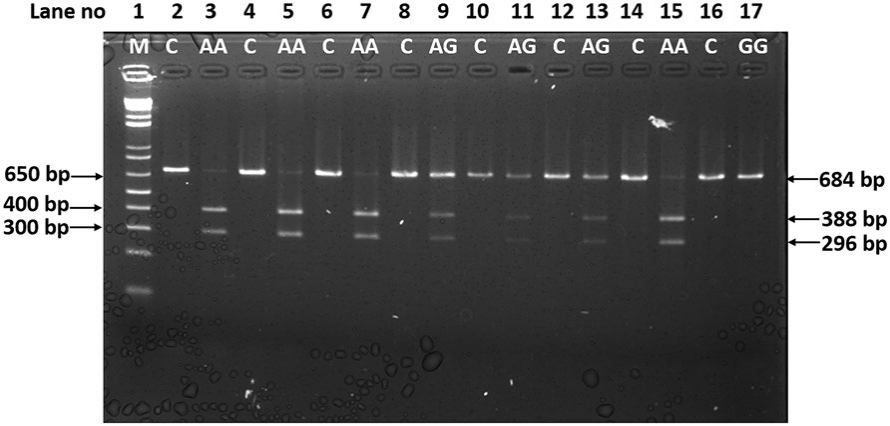
Genotyping of samples by RFLP analysis. Lane 1(M): 1 kb plus DNA ladder, lanes 2, 4, 6, 8, 10, 12, 14 and 16; Undigested PCR product (C = control); lane 3, 5, 7 and 15; AA Homozygous genotype, lanes 9, 11 and 13; AG Heterozygous genotypes and lane 17; GG Homozygous genotype

Briefly, the region comprising rs7756992 polymorphism was amplified with the following primers: Forward 5ʹ-TTGATTGTAAAGACTGGGTCTCA-3ʹ; Reverse 5ʹ-GAACGAAGGCAAATAAATTCAA-3ʹ. The PCR cycles were as follows: 5 min at 95 °C, followed by 35 cycles of 1 min at 95 °C, 1 min at 50 °C, and 1 min at 72 °C. The final extension was for 7 min at 72 °C. Subsequently, 7 µL of the 684 bp amplified product was digested with the restriction enzyme BglII (neb) for 1 h at 37 °C. After digestion, amplicons were subjected to 2% agarose gel electrophoresis. The appearance of two bands of 388 bp and 296 bp was indicative of the AA genotype, whereas 684 bp digestion products indicated the GG genotype. When heterozygous genotypes exist, three bands were observed (Fig. 1).

### DNA sequencing

**Fig. 2:**
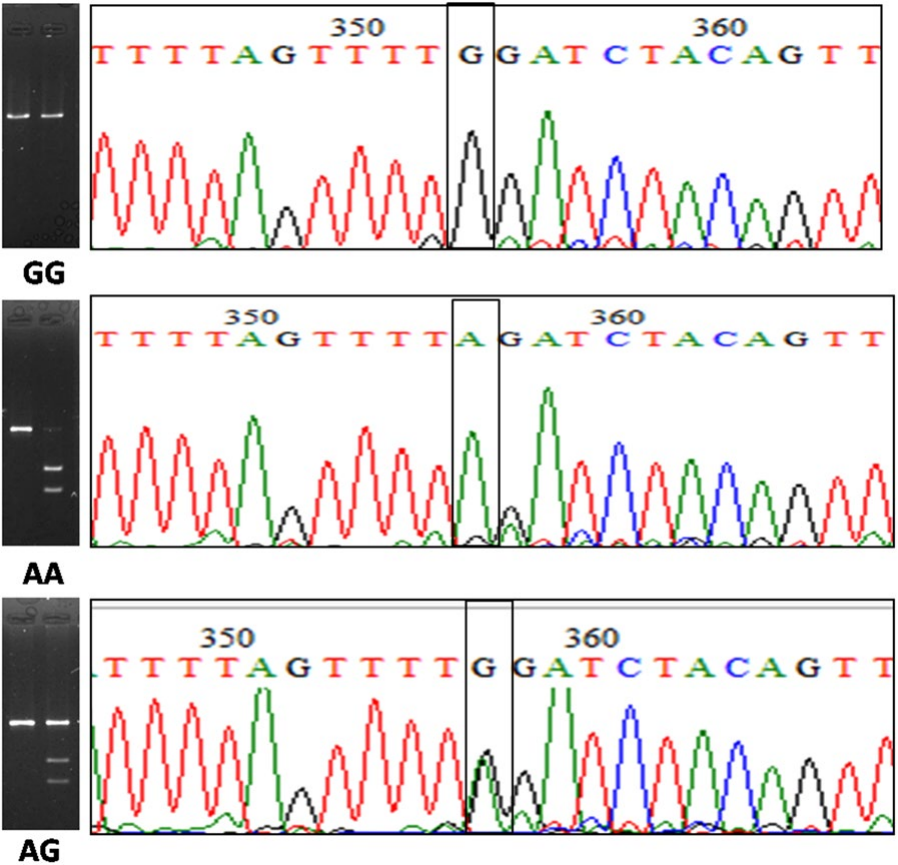
Confirmation of the RFLP results by sequencing

PCR RFLP genotyping results were validated by sequencing randomly selected samples (Fig. 2). The amplified *CDKAL1* locus was sequenced by Sanger’s di-deoxy chain terminating method using a sequencing kit (BigDye Terminator v3.1, Applied Biosystems, Foster City, CA, USA). Sequences were analyzed to determine the genotype using sequencing analysis software, version 5.2 (Applied Biosystems, Foster City, CA, USA).

### TaqMan™ allelic discrimination assay

**Fig. 3:**
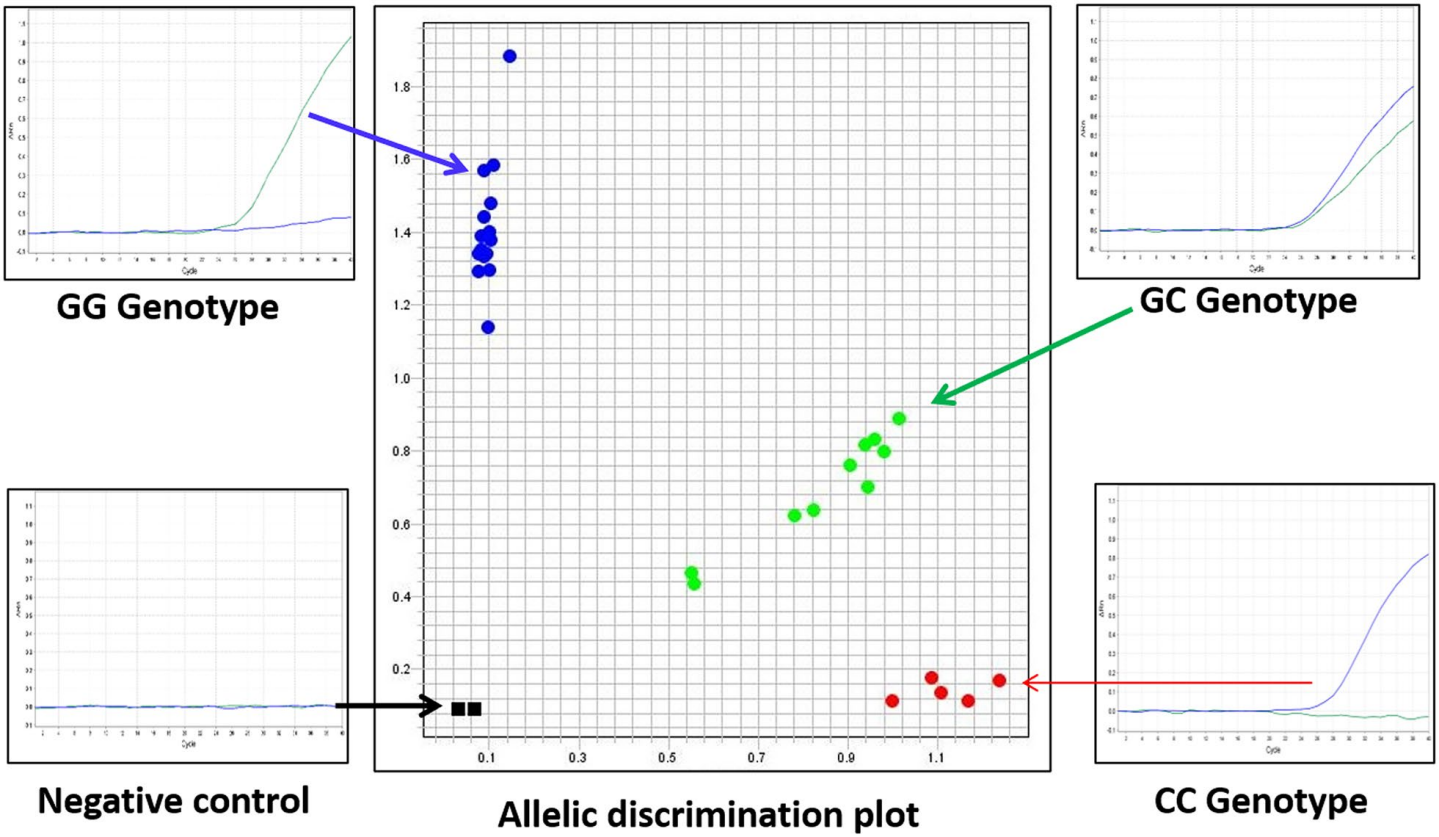
An allele discrimination plot from a single run of 30 samples containing both cases and controls and representative amplification plots for each genotype, as well as a negative control. Blue, green and red dots denoted GG, GC and CC genotypes respectively; black squares denoted negative controls

All samples were genotyped for rs7754840 using allelic discrimination assay on a QuantStudio™ 5 Real-Time PCR System for human identification (Applied Biosystems, USA) with predesigned TaqMan™ SNP Genotyping Assay, human (assay ID: C_29246232_10). As a quality standard previously sequenced three positives have been randomly included (one homozygous wild-type allele carrier, one heterozygous, and one homozygous risk allele carrier) and three negative (all components excluding DNA) controls in each run. QuantStudio Design and analysis software v 1.4.3 was used to analyze real-time data (Fig. 3). 20% samples were repeated and no differences were found.

### Statistical analyses

Normality of the random variables was checked by visual inspection of histograms using R statistical software version 4.0.3. Student’s t-tests were used to compare the ages, BMI, systolic blood pressure, diastolic blood pressure, and plasma glucose levels (FPG, OPG, and TPG) between the GDM and control groups while the family history of diabetes and gravidity between these groups were tested using the Chi-square test. Numerical variables were expressed as mean ± standard deviation of the mean (mean ± SD). On the other hand, categorical variables were expressed in numbers (n) and percentages (%). Hardy–Weinberg equilibrium (HWE) was tested using Pearson’s chi-squared (χ^2^) test with a threshold of *P* > 0.05 in cases and controls separately. Risk factors of GDM were detected by multivariate logistic regression. The general association of genotypes with GDM was assessed with multivariate logistic regression analysis under codominant, dominant, recessive, overdominant, and log-additive models and adjusted for gravidity and family history of diabetes by using SNPStats [32]. The best-fitting model with multiple variables was chosen by stepwise addition of prospective confounding variables using Akaike’s Information Criterion (AIC) and Bayesian Information Criterion (BIC) values which are criteria of goodness of fit. The association of haplotypes of rs7756992 and rs7754840 with GDM was carried out SNPStats [32]. The statistical power was calculated using the GAS power calculator [33].

## Results

### General characteristics of the study subjects and risk factors of GDM

The study included 468 subjects classified into 212 pregnant women with GDM and 256 control subjects. Since it was a time-bound study, sample collection was stopped after the stipulated time period. Their age ranged from 18 to 44 (years). Participants with GDM had significantly higher age (27.58 ± 4.59 vs. 25.42 ± 4.58, *P* < 0.0001), BMI (26.64 ± 4.15 vs. 25.29 ± 3.92, *P* = 0.0003), plasma glucose levels (FPG: 5.15 ± 0.47 vs. 4.31 ± 0.48, *P* < 0.0001; OPG: 9.77 ± 1.65 vs. 7.49 ± 1.26, *P* < 0.0001 and TPG: 8.25 ± 1.49 vs. 6.4 ± 1.06, *P* < 0.0001); higher percentage of positive family history of diabetes (46.23% vs. 30. 0.85%, *P* = 0.0006), multigravida (62.74% vs. 53.52%, *P* = 0.04) and lower percentage of primigravida (35.85% vs. 46. 48%, *P* = 0.02) than those in controls. There is no significant difference between systolic (109.27 ± 11.75 vs. 108.94 ± 11.91, *P* = 0.69) and diastolic (70.51 ± 9.18 vs. 69.25 ± 9.05, *P* = 0.12) blood pressures between these two groups. As odds of age (OR = 1.1, 95% CI 1.06–1.15, *P* < 0.001) and BMI (OR = 1.08, 95% CI 1.03–1.14, *P* = 0.002) were very close to 1 they could not be risk factors of GDM. On the other hand risk of GDM was higher in gravidity (OR = 1.53, 95% CI 1.05–2.23, *P* = 0.03) and family history of diabetes (OR = 1.93, 95% CI 1.32–2.83, *P* < 0.001), so they are the confounders for which adjustments were carried out.

### Genotype frequency and association of *CDKAL1* gene variants with GDM

Genotyping for rs7756992 and rs7754840 polymorphisms of *CDKAL1* gene revealed that genotype distribution of these two SNPs differed in individuals with and without GDM (Table 1). Frequencies of AG (53% vs. 45%) and GG (10% vs. 7%) genotype of rs7756992 were higher in the GDM group whereas the control group comprised a higher proportion of AA (48% vs. 37%) genotypes. In case of rs7754840 polymorphism CC (9% vs. 5%) genotype is higher in the GDM group and GG (55% vs. 51%) in the control group whereas the frequency of CG genotype is almost equal (GDM-40.1% vs. control-39.8%) in both groups. Genotype distributions of cases and controls of the last-mentioned and controls of former were consistent with Hardy–Weinberg equilibrium (HWE) (Table 1) and could be used for the following analysis. Departure from HWE resulted in genotype distribution of rs7756992 in cases which indicates that there may be an association of this SNP with GDM.

**Table 1:** Genotype and allele frequency of *CDKAL1* gene variants (rs7756992 and rs7754840) in the study subjects

SNP	Genotype/allele	Control (%)	GDM (%)
rs7756992	AA	123 (48)	78 (36.8)
	AG	114 (44.5)	113 (53.3)
	GG	19 (7.4)	21 (9.9)
	A	360 (70.31)	269 (63.44)
	G	153 (29.65)	155 (36.56)
HWE	Chi-square (χ^2^)	1.14	4.71
	*P* value	0.29	0.03
rs7754840	GG	141 (55.1)	107 (50.5)
	GC	102 (39.8)	85 (40.1)
	CC	13 (5.1)	20 (9.4)
	G	384 (75)	299 (71)
	C	128 (25)	125 (29)
HWE	Chi-square (χ^2^)	1	0.27
	*P* value	0.317	0.603

**Fig. 4:**
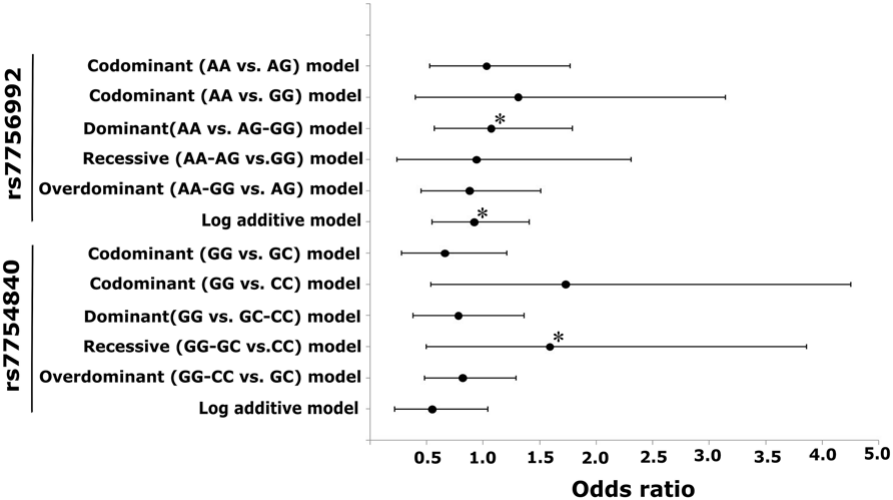
Associations of rs7756992 (AA/AG/GG) and rs7754840 (GG/GC/CC) with GDM under different genetic models adjusted for family history of diabetes and gravidity with odds ratios shown by closed circles and whiskers representing the 95% confidence intervals

Association of these genetic variants with GDM was tested under codominant, dominant, recessive, overdominant and log additive models (Additional file 1: Table S1, S2). Our analyses revealed significant (*P* < 0.05) association of rs7756992 with GDM patients under codominant [AA vs. AG (OR = 1.56, 95% CI 1.06 to 2.30), AA vs. GG (OR = 1.74, 95% CI 0.88 to 3.45), *P* = 0.047] dominant (OR = 1.59, 95% CI 1.10 to 2.30, *P* = 0.014) and log additive (OR = 1.42, 95% CI 1.06 to 1.90, *P* = 0.019) models. Odds of having GDM is also higher in both recessive (OR = 1.37, 95% CI 0.72 to 2.62, *P* = 0.34) and over dominant (OR = 1.42, 95% CI 0.99 to 2.05, *P* = 0.059) models though *P* value is > 0.05 in both (Additional file 1: Table S1). Adjustment for gravidity and family history of diabetes results significant associations of this variant with GDM under dominant (*P* = 0.02) and log additive (*P* = 0.022) models but significance of codominant model increased from *P* = 0.047 to *P* = 0.062 (Additional file 1: Table S1) even though the odd remained considerably higher (OR = 1.81) than that of reference genotype (Fig. 4).

On the other hand no significant association was found between rs7754840 and GDM under any genetic model [co-dominant model {(GG vs. CG): OR = 1.10, 95% CI 0.75 to 1.61; (GG vs. CC): OR = 2.03, 95% CI 0.97 to 4.26} *P* = 0.17; dominant model: OR = 1.20, 95% CI 0.84 to 1.73, *P* = 0.32; recessive model: OR = 1.95, 95% CI 0.94 to 4.01, *P* = 0.067; overdominant: OR = 1.01, 95% CI 0.70 to 1.47, *P* = 0.96 and log-additive: OR = 1.26, 95% CI 0.94 to 1.69, *P* = 0.12] (Additional file 1:Table S2). When adjusted for confounding covariates the odds of having GDM with CC genotype increased to 2.23 with a significance level of 95% CI from 1.04 to 4.75 though *P* value remained greater than 0.05. Impressively this adjustment results significant (*P* = 0.047) association between this SNP with GDM under recessive model with an OR of 2.09 (95% CI 1.00 to 4.36) and an increase in odds under overdominant model and vice versa under log additive model with a tiny change in *P* values. There was no notable change observed under dominant model (Fig. 4).

### Association of risk alleles of studied SNPs with GDM

The frequency of the G allele of rs7756992 was higher in GDM whereas that of the A allele was higher in control (GDM vs. control, G-allele: 37% vs. 30%; A-allele 63% vs. 70%) (Table 1). In presence of the G allele, the odds of having GDM increase significantly (*P* = 0.029) by 1.36-fold with a 95% CI of 1.03 to 1.78. Though the frequency of the C allele of rs7754840 was also higher in the GDM group (29% vs. 25%) the effect of this allele on the susceptibility of GDM is insignificant (OR = 1.25, 95% CI 0.94 to 1.67, *P* = 0.125).

### Association of *CDKAL1* gene variants with the family history of diabetes and gravidity

**Fig. 5:**
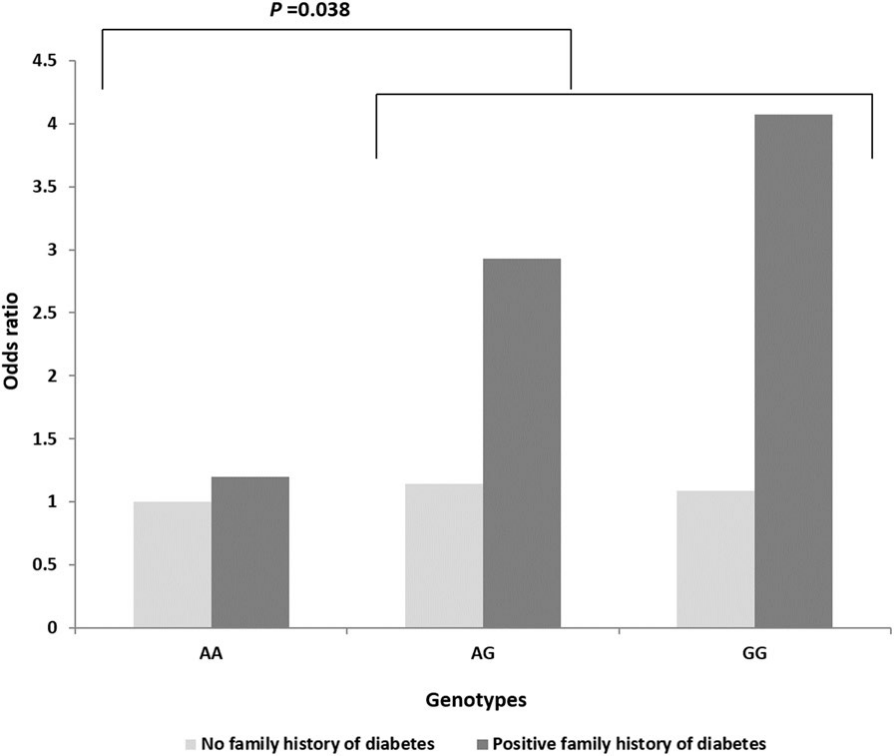
Correlation between GDM risk with cumulation of *CDKAL1* rs7756992 polymorphism and family history of diabetes

**Fig. 6:**
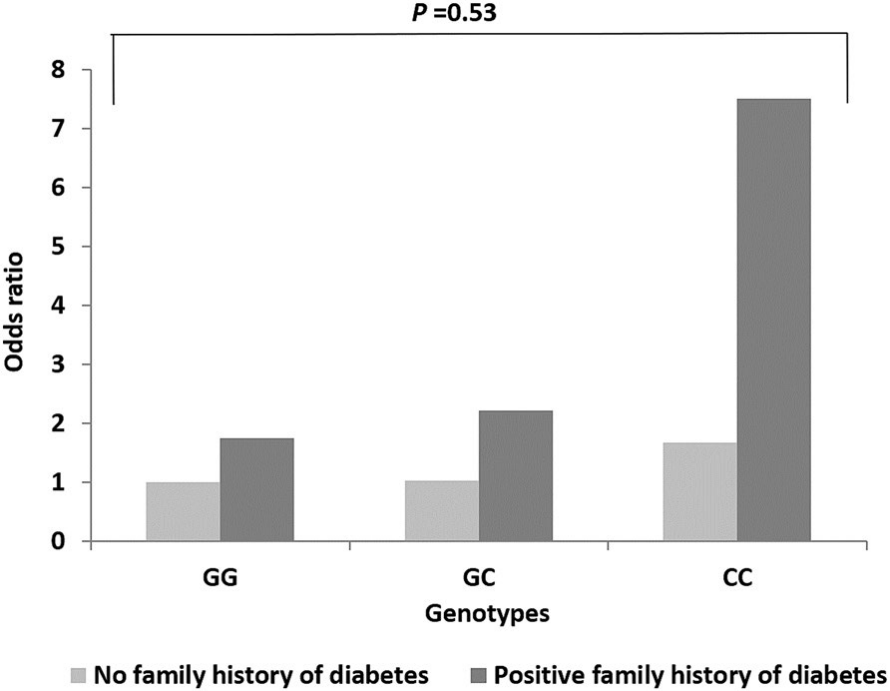
Correlation between GDM risk with cumulation of *CDKAL1* rs7754840 polymorphism and family history of diabetes

Logistic regression analysis was carried out to check the relationship between the variants with the family history of diabetes under two genetic models (Additional file 1: Table S3). This cross-classification interaction with rs7756992 and rs7754840 results 1.18 (95% CI 0.65 to 2.14) to 4.02 (95% CI 1.33 to 12.09) (Fig. 5) and 1.72 (95% CI 1.02 to 2.89) to 7.42 (95% CI 1.55 to 35.57) (Fig. 6) folds increased in odds of having GDM with a positive family history of T2DM respectively. A significant *P* value (0.038) was obtained only from the interaction of rs7756992 with GDM (Fig. 5).

**Fig. 7:**
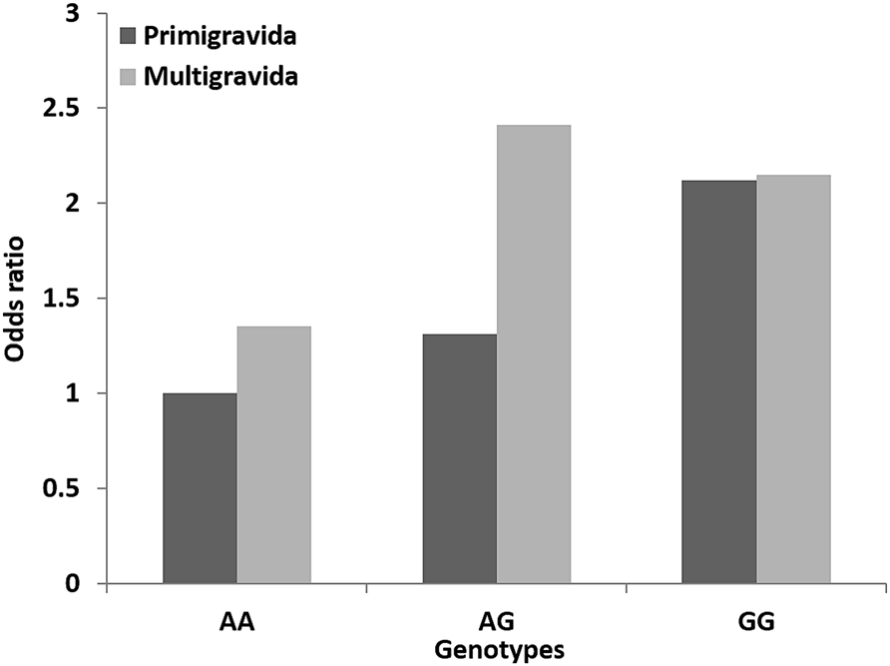
Correlation between GDM risk with cumulation of *CDKAL1* rs7756992 polymorphism and gravidity

**Fig. 8:**
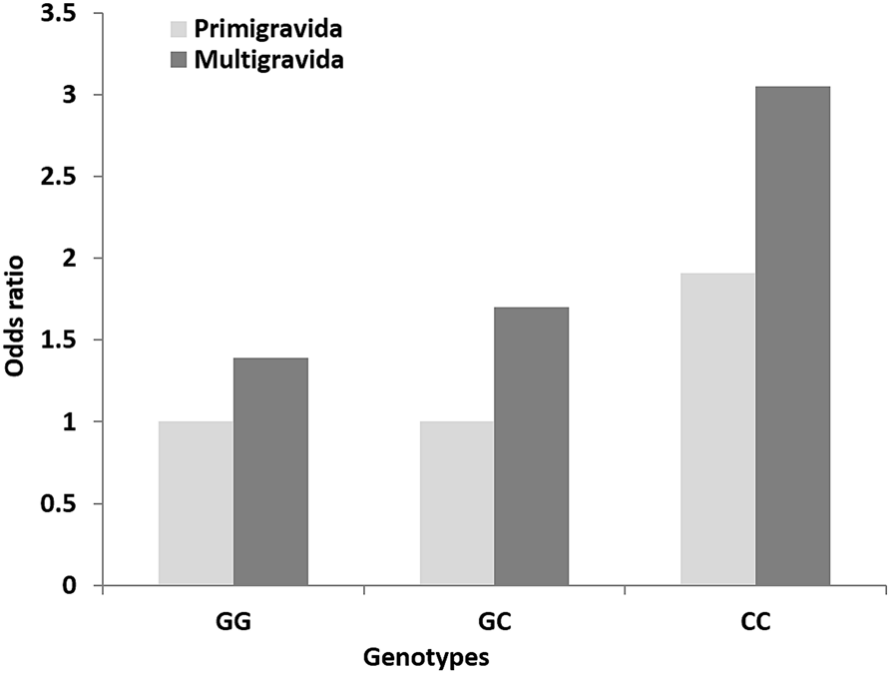
Correlation between GDM risk with cumulation of *CDKAL1* rs7754840 polymorphism and gravidity

In multigravida women, there was a noticeable increase observed in the odds of having GDM compared to primigravida in the case of rs7754840 with odds varied from 1.39 (95% CI 0.83–2.32) to 3.05 (95% CI 1.12–8.30) under the codominant and dominant model (Fig. 8). For AA and AG genotype of rs7756992 odd ratios increased in multigravida than that of primigravida under both models whereas for GG genotype risk of having GDM increased by more than two folds [OR = 2.09, (95% CI 0.79–5.49) and 2.11 (0.77–5.77)] in both strata (Fig. 7 and Additional file 1: Table S4).

### The association of haplotype of rs7756992 and rs7754840 with GDM

Taking the common rs7754840/rs7756992 GA haplotype as reference (OR = 1.00), multivariate analysis adjusted for gravidity and family history of diabetes confirmed the association of rs7754840 C- and rs7756992 G-allele containing (CG) haplotype with GDM, thus conferring significant (*P* = 0.032) disease susceptibility with an odd of 1.43 (1.03–1.98) (Table 2).

**Table 2:** The association of haplotype of rs7756992 and rs7754840 with GDM

Sl.	rs7754840	rs7756992	Frequency	OR (95% CI)	*P*-value^a^
1	G	A	0.6417	1.00	
2	C	G	0.243	1.43 (1.03–1.98)	0.032
3	G	G	0.0857	1.40 (0.84–2.33)	0.19
4	C	A	0.0296	1.15 (0.50–2.67)	0.74

^a^Adjusted for gravidity and family history of diabetes

## Discussion

Among others, *CDKAL1* is one of the first set of T2DM susceptibility genes identified by genome-wide association (GWA) and other studies [19, 20, 34–38]. Variants of this gene rs7756992 A/G and rs7754840 C/G included in our study were previously found to be associated with T2DM among different Asian, European, and American populations [19, 20, 26, 28, 29, 39–43]. The association of these genetic variants with GDM was also studied in a number of populations as this disease may have a common genetic background with T2DM [27, 44, 45]. Recently some studies on rs7756992 and rs7754840 reported significant association in Asian populations [26, 28, 46, 47] whereas in some populations there was no association found [45, 48]. Nevertheless, no report is available on the association of *CDKAL1* rs7756992 and rs7754840 SNPs with GDM in Bangladeshi women.

In our study confirmation of the GDM cases was crucial and challenging. Insulin resistance mediated by placental hormones increases GDM as the pregnancy advances so testing too early for GDM may not be of help in some patients. During the second trimester (13–28 weeks of gestation) of pregnancy insulin resistance increases and glucose levels rise in women unable to produce enough insulin to adopt this resistance. Also, performing tests too late in the third trimester limits the time of metabolic interventions; hence GDM screening is usually preferred at 24–28 weeks of gestation [49]. Taking these into consideration case samples were collected irrespective of the trimester, and individuals having normal plasma glucose levels at the earlier stage of pregnancy were further tested for confirmation at 24–28th weeks of gestation.

GAS power calculator [33] was used to detect the power of study and considering the total sample size (both cases and controls) studied (n = 468), genotype relative risks (1.74 and 2.03) for rs7756992 and rs7754840, the prevalence of GDM (0.366) in Bangladeshi population [3], disease allele frequency obtained (0.36 and 0.29), the significance level equal to 0.05 we had almost 98% power for both SNPs which indicates that the study sample size had sufficient power to detect the association.

The genotype distributions of the target SNPs (rs7756992 and rs7754840) were in concordance with HWE in the control group which indicates that there is no selection bias, population stratification, and genotyping errors [50] in the study population. In addition to the control, cases should also be evaluated to avoid eliminating important SNPs that could potentially be causal SNPs of a common disease [51]. Therefore, the distribution of genotypes of both target SNP cases has been assessed for HWE and rs7756992 showed significant (*P* = 0.03) departure from the equilibrium which is indicative of a strong association of this SNP with GDM. In accordance with the positive associations that have been reported in the Asian population [26, 52] our data also shows a significant association of rs7756992 with GDM. The effect size of the rs7756992 G allele (OR = 1.36), is close in magnitude to those reported for Asians (OR = 1.41), and south Indians (OR = 1.45). In our study, association analyses were performed under several genetic models namely codominant, dominant, recessive, overdominant, and log additive to avoid possible biases in finding and reporting significant associations [53] and three of them-codominant (*P* = 0.047), dominant (*P* = 0.014) and log additive (*P* = 0.019) models revealed significant (*P* < 0.05) association (Additional file 1: Table S1) between this SNP with GDM. Adjustments for gravidity and family history of diabetes result in no change in the significant association of rs7756992 with GDM under dominant and log additive models. After adjustment, GG genotype showed a higher odd ratio (1.81) whereas the odd of AG genotype remained comparable with an insignificant *P* value (Fig. 4).

Though there is no significant association observed between rs7754840 and GDM risk in our study, the homozygous risk allele of this SNP results in higher odds of having GDM compared to that of rs7756992. Our findings are consistent with a Chinese and Egyptian study where no significant association was found [45, 54], while studies in Caucasian, Korean and South Indian populations showed significant associations [26, 52, 55] indicating that the common susceptibility loci rs7754840 in *CDKAL1* may be not associated with GDM in our population. These variations in association could be due to differences in ethnicities, sample sizes, and diagnostic criteria for GDM. However, after adjusted for gravidity and family history of diabetes, the recessive model revealed a significant association (*P* = 0.047) of this variant with GDM (Fig. 4).

Systolic and diastolic blood pressures were indistinguishable between control and GDM groups whereas significant differences were observed in age, BMI, gravidity, and family history of diabetes. Consequently, the latter were the prospective confounders that should be adjusted for in the subsequent analysis. To detect actual confounding factors multivariate logistic regression was carried out and from the value of odds ratios, risk factors were determined. Among the variables of this study age, BMI, gravidity, and family history of diabetes were selected as potential confounders [56]. Multivariate logistic regression analysis revealed gravidity (OR = 1.53) and family history of diabetes (OR = 1.93) as confounders in this study as they have a higher risk for GDM. In contrast to other types of biases, confounding can be controlled by adjusting for it after completion of a study using stratification [56], hence the population of this study was divided into strata or subgroups according to levels of the confounding factors. Relative risk analysis for each stratum was carried out for control and GDM groups by cross classification interactions under different models (Additional file 1: Table S3 and S4).

The cumulative effect of *CDKAL1* rs7756992 polymorphism and positive family history of diabetes significantly increased the risk of GDM by 2.7-fold under the dominant genetic model (Additional file 1: Table S3). The AG and GG genotypes of this SNP increased the odds of GDM by 2.5 and 3.7 folds respectively whereas none of these genotypes provides any risks to individuals with a negative family history of diabetes (Fig. 5). Mansoori et al. suggested an association between the CC genotype of the rs7754840 polymorphism and people with late-onset T2DM [57]. In our analysis not only the CC genotype of this SNP increased the odds of having GDM 4.5 times but also the GG genotype increased it 1.72 times in women with a positive family history of diabetes (Fig. 6). These genetic predispositions of both variants may be a valuable marker for differentiating pregnant women with an elevated risk of GDM who could then be subjected to an earlier routine screening for GDM as well as lifestyle intervention before and after pregnancy to avoid the onset of GDM and T2DM respectively. In addition, new research avenues will open up in the search for cost-effective technology for early routine screening for GDM.

Multigravidity itself increased the risk of GDM compared to that in primigravida women i.e., increased odds of having GDM in presence of homozygous reference alleles of both SNPs (1.33-fold for AA genotype of rs7756992 and 1.39-fold for GG genotype of rs7754840). The presence of multigravidity and heterozygous risk alleles of rs7756992 (AG) and rs7754840 (GC) collectively increased the odds of GDM by 1.80 and 1.70 folds respectively whereas only the homozygous risk allele (CC) of rs7754840 increased the odds by 1.59-fold (Figs. 7 and 8). The homozygous risk allele of rs7756992 increased the risk of GDM by more than two folds in both primi and multigravida women (Fig. 7). Insignificant *P* values and wider CI values of different outcomes could be explained by the small sample size in each subgroup of both confounders (Additional file 1: Table S3, S4).

The haplotype analysis of these SNPs revealed significant association of haplotype containing both alleles with GDM susceptibility that provides further evidence for the resulting association of this study.

The findings of the current study might be specific to GDM patients who lived in greater Dhaka city and used to be with the city lifestyle as they were recruited from Dhaka city and nearby regions such as Narayanganj and Gazipur. This may also explain the nature of the association between *CDKAL1* rs7756992 and rs7754840 SNPs with GDM in this study. Though several risk factors are counted here in this study but other GDM associated factors such as food intake and physical activity could not be considered due to the unavailability of information. Therefore, interactions between lifestyle factors and these genetic variants in terms of GDM remain unknown. Furthermore, rs7756992 and rs7754840 are located in the intron of *CDKAL1*; thus, the relationships between these SNPs and genes and how they modulate GDM risk are largely unknown.

## Conclusions

**Fig. 9:**
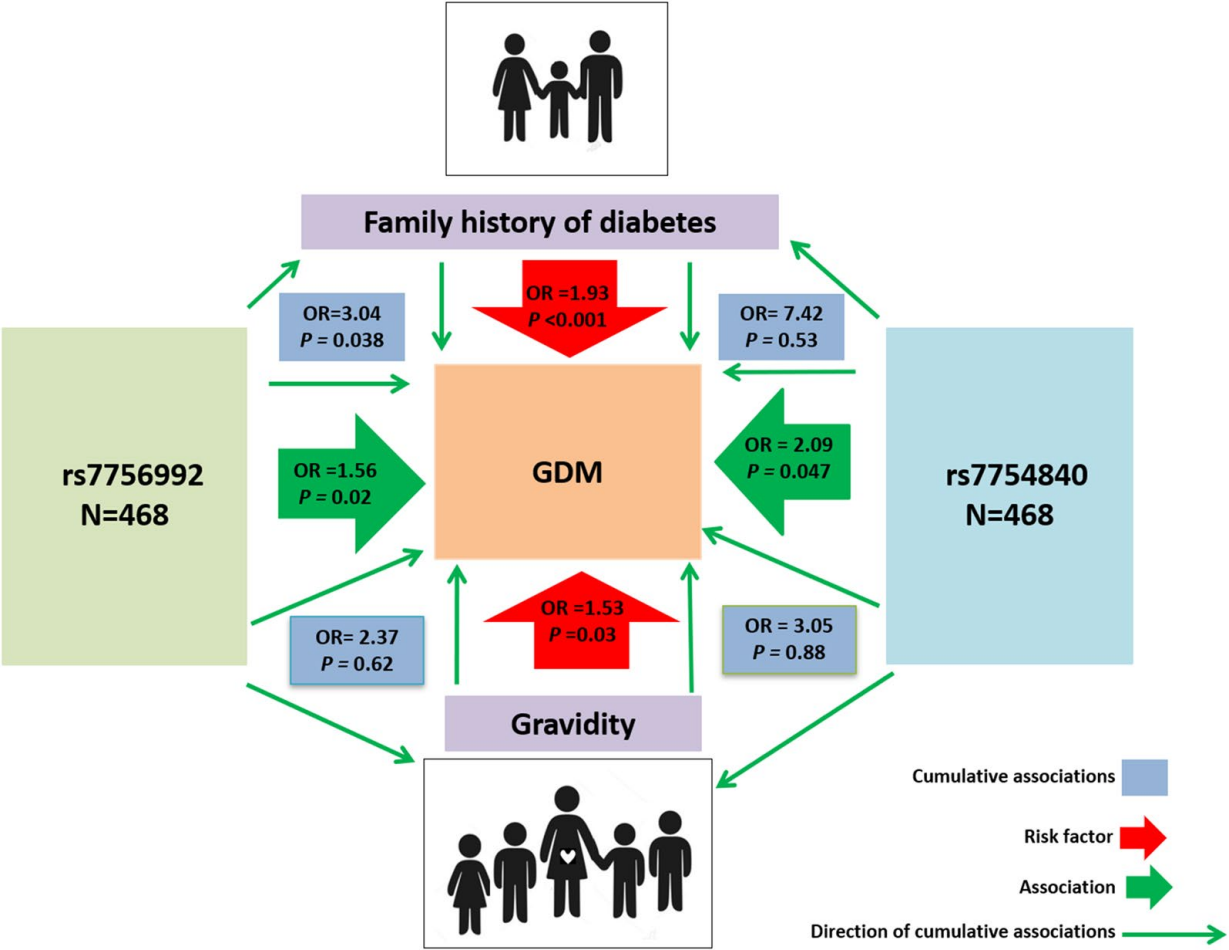
Cumulation of genetic association of GDM with *CDKAL1* rs7756992 and rs7754840 polymorphisms along with confounders

In conclusion, our study provides evidence that rs7756992 and rs7754840 are either significantly associated or increased the risk of GDM in pregnant Bangladeshi women, emphasizing the importance of these potentially functional variants in GDM development (Fig. 9). The rs7754840 is not only significantly associated with but also results in higher odds of having GDM compared to rs7756992. The underlying mechanisms are needed to be elucidated through functional investigations for the identification of causal loci and genes. The sample size in this study yielded power to identify significant associations between GDM and variants in the *CDKAL1* gene, but stratification for confounding factors decreased the number of samples in each stratum that eventually resulted in insignificant associations with variants in some strata. Furthermore, large studies including other ethnic populations are needed to further replicate our findings.

## Supplementary Information

**Additional file 1**: **Table S1. **Association of rs7756992 with GDM under different genetic models.** Table S2. **Association of rs7754840 with GDM under different genetic models.** Table S3. **Cross classification interaction table of *CDKAL1* variants (rs7756992 and rs7754840) and family history of T2DM.** Table S4. **Cross classification interaction table of *CDKAL1* variants (rs7756992 and rs7754840) and gravidity.

## Data Availability

All data generated or used during the study are available from the corresponding author on reasonable request: Dr. Md. Salimullah, E-mail: salim2969@gmail.com.
