# Supplementary material for: CDKAL1 gene rs7756992 A/G and rs7754840 G/C polymorphisms are associated with gestational diabetes mellitus in a sample of Bangladeshi population: implication for future T2DM prophylaxis
Source: Diabetol Metab Syndr. 2022 Jan 28;14:18. doi: 10.1186/s13098-021-00782-w (PMC8796445; doi:10.1186/s13098-021-00782-w)
Supplement: Supplementary file 1 — Additional file 1: Table S1. Association of rs7756992 with GDM under different genetic models. Table S2. Association of rs7754840 with GDM under different genetic models. Table S3. Cross classification interaction table of CDKAL1 variants (rs7756992 and rs7754840) and family history of T2DM. Table S4. Cross classification interaction table of CDKAL1 variants (rs7756992 and rs7754840) and gravidity. [file 13098_2021_782_MOESM1_ESM.docx]

**Table S1: Association of rs7756992 with GDM under different genetic models**

| **Model** | **Control (%)** | **GDM (%)** | **OR (95% CI)** | ***P* value** | **OR (95% CI) ^a^** | ***P* value ^a^** |
| --- | --- | --- | --- | --- | --- | --- |
| **Codominant**  A/A  A/G  G/G | 123 (48) | 78 (36.8) | 1.00 | **0.047** | 1.00 | 0.062 |
|  | 114 (44.5) | 113 (53.3) | **1.56 (1.06-****2.30)** |  | **1.52 (1.03-2.26)** |  |
|  | 19 (7.4) | 21 (9.9) | 1.74 (0.88-3.45) |  | 1.81 (0.90-3.64) |  |
| **Dominant**  A/A  A/G-G/G | 123 (48) | 78 (36.8) | 1.00 | **0.014** | 1.00 | **0.02** |
|  | 133 (52) | 134 (63.2) | **1.59 (1.10-2.30)** |  | **1.56 (1.07-2.29)** |  |
| **Recessive**  A/A-A/G  G/G | 237 (92.6) | 191 (90.1) | 1.00 | 0.34 | 1.00 | 0.28 |
|  | 19 (7.4) | 21 (9.9) | 1.37 (0.72-2.62) |  | 1.45 (0.74-2.81) |  |
| **Overdominant**  A/A-G/G  A/G | 142 (55.5) | 99 (46.7) | 1.00 | 0.059 | 1.00 | 0.095 |
|  | 114 (44.5) | 113 (53.3) | 1.42 (0.99-2.05) |  | 1.38 (0.95-2.00) |  |
| **Log-additive** | --- | --- | **1.42 (1.06-1.90)** | **0.019** | **1.42 (1.05-1.91)** | **0.022** |

**^a^ adjusted for gravidity and family history of diabetes**

**Table S2: Association of rs7754840 with GDM under different genetic models**

| **Model** | **Control (%)** | **GDM (%)** | **OR (95% CI)** | ***P* value** | **OR (95% CI) ^a^** | ***P* value ^a^** |
| --- | --- | --- | --- | --- | --- | --- |
| **Codominant**  G/G  G/C  C/C | 141 (55.1) | 107 (50.5) | 1.00 | 0.17 | 1.00 | 0.11 |
|  | 102 (39.8) | 85 (40.1) | 1.10  (0.75-1.61) |  | 1.16  (0.78-1.72) |  |
|  | 13 (5.1) | 20 (9.4) | 2.03  (0.97-4.26) |  | **2.23**  **(****1.04-4.75)** |  |
| **Dominant**  G/G  G/C-C/C | 141 (55.1) | 107 (50.5) | 1.00 | 0.32 | 1.00 | 0.2 |
|  | 115 (44.9) | 105 (49.5) | 1.20 (0.84-1.73) |  | 1.28  (0.88-1.86) |  |
| **Recessive**  G/G-G/C  C/C | 243 (94.9) | 192 (90.6) | 1.00 | 0.067 | 1.00 | 0.047 |
|  | 13 (5.1) | 20 (9.4) | 1.95 (0.94-4.01) |  | **2.09**  **(1.00-4.36)** |  |
| **Overdominant**  G/G-C/C  G/C | 154 (60.2) | 127 (59.9) | 1.00 | 0.96 | 1.00 | 0.8 |
|  | 102 (39.8) | 85 (40.1) | 1.01  (0.70-1.47) |  | 1.05  (0.72-1.54) |  |
| **Log-additive** | --- | --- | 1.26 (0.94-1.69) | 0.12 | 1.33  (0.98-1.79) | 0.065 |

**^a^ adjusted for gravidity and family history of diabetes**

**Table S3: Cross classification interaction table of *CDKAL1* variants (rs7756992 and rs7754840) and family history of T2DM**

| **SNP** | **Models** | **Family history of T2DM (n=467)** | | | | | | **Interaction**  ***P* value** |
| --- | --- | --- | --- | --- | --- | --- | --- | --- |
|  |  | **No** | | | **Yes** | | |  |
|  |  | **Control** | **GDM** | **OR**  **(95% CI)** | **Control** | **GDM** | **OR**  **(95% CI)** |  |
| rs7756992 | **Codominant**  A/A  A/G  G/G | 82 | 49 | 1.00 | 41 | 29 | 1.18  (0.65-2.14) | 0.1 |
|  |  | 81 | 55 | 1.14  (0.69-1.86) | 33 | 57 | **2.89**  **(1.66-5.04)** |  |
|  |  | 14 | 9 | 1.08  (0.43-2.67) | 5 | 12 | **4.02**  **(1.33-****12.09)** |  |
|  | **Dominant**  A/A  A/G-G/G | 82 | 49 | 1.00 | 41 | 29 | 1.18  (0.65-2.14) | **0.038** |
|  |  | 95 | 64 | 1.13  (0.70-1.81) | 38 | 69 | **3.04**  **(1.79-5.17)** |  |
| rs7754840 | **Codominant**  G/G  G/C  C/C | 94 | 57 | 1.00 | 47 | 49 | **1.72**  **(****1.02-****2.89)** | 0.53 |
|  |  | 72 | 45 | 1.03  (0.63-1.69) | 30 | 40 | **2.20**  **(1.24-3.91)** |  |
|  |  | 11 | 11 | 1.65  (0.67-4.05) | 2 | 9 | **7.42**  **(****1.55-****35.57)** |  |
|  | **Dominant**  G/G  G/C-C/C | 94 | 57 | 1.00 | 47 | 49 | **1.72**  **(1.02-2.89)** | 0.48 |
|  |  | 83 | 56 | 1.11  (0.69-1.78) | 32 | 49 | **2.53**  **(1.45-4.39)** |  |

**Table S4: Cross classification interaction table of *CDKAL1* variants (rs7756992 and rs7754840) and gravidity**

| **SNP** | **Models** | **Gravidity(n=465)** | | | | | | **Interaction**  ***P* value** |
| --- | --- | --- | --- | --- | --- | --- | --- | --- |
|  |  | **Primigravida** | | | **Multigravida** | | |  |
|  |  | **Control** | **GDM** | **OR**  **(95% CI)** | **Control** | **GDM** | **OR**  **(95% CI)** |  |
| rs7756992 | **Codominant**  A/A  A/G  G/G | 55 | 29 | 1.00 | 67 | 47 | 1.33  (0.74-2.39) | 0.6 |
|  |  | 52 | 36 | 1.31  (0.71-2.44) | 61 | 76 | **2.36**  **(1.35-4.14)** |  |
|  |  | 10 | 11 | 2.09  (0.79-5.49) | 9 | 10 | 2.11  (0.77-5.77) |  |
|  | **Dominant**  A/A  A/G-G/G | 56 | 29 | 1.00 | 67 | 47 | 1.35  (0.76-2.43) | 0.62 |
|  |  | 63 | 47 | 1.44  (0.80-2.59) | 70 | 86 | **2.37**  **(1.37-4.10)** |  |
| rs7754840 | **Codominant**  G/G  G/C  C/C | 64 | 39 | 1.00 | 77 | 65 | 1.39  (0.83-2.32) | 0.88 |
|  |  | 49 | 30 | 1.00  (0.55-1.84) | 53 | 55 | 1.70  (0.98-2.95) |  |
|  |  | 6 | 7 | 1.91  (0.60-6.11) | 7 | 13 | **3.05**  **(1.12-8.30)** |  |
|  | **Dominant**  G/G  G/C-C/C | 64 | 39 | 1.00 | 77 | 65 | 1.39  (0.83-2.32) | 0.61 |
|  |  | 55 | 37 | 1.10  (0.62-1.96) | 60 | 68 | **1.86**  **(1.10-3.15)** |  |
